# Efficiency of utilization of dietary energy for milk production in lactating crossbred cattle (*Bos Indicus*)

**Published:** 2012

**Authors:** Debashis Saha, Radhe Shyam Gupta, Ramesh Pratap Singh Baghel, Ankur Khare

**Affiliations:** Department of Animal Nutrition, College of Veterinary Science and Animal Husbandry, Madhya Pradesh Pashu Chikitsa Vigyan Vishwavidyalaya, Jabalpur, India

**Keywords:** Diammonium phosphate, Dietary energy, Cattle, Milk yield, Gross energy

## Abstract

The present study was conducted on efficiency of utilization of dietary energy for milk production in lactating crossbred cattle. 18 lactating crossbred cattle of early to mid-lactation, approximate body weight (375.39±23.43 kg), milk yield, parity and stage of lactation were divided into three groups of six animals each and were fed 0, 50 and 100% diammonium phosphate (DAP) in the mineral mixture of concentrates for 120 days. The chaffed mixed roughage (berseem + wheat straw) and concentrate mixture was fed to supply about nearly 18:82 concentrate to roughage ratio on dry matter basis. Tap water was available to the animals twice daily. A metabolism trial of seven days was conducted at the end of experiment to study digestibility of organic nutrients and balances of energy. DAP did not affect the nutrient intake, body weight changes, digestibility of Dry matter (DM), Crude protein (CP), Ether extract (EE), Crude fiber (CF), Nitrogen free extract (NFE) and daily milk yield. It was concluded that the at 46.07 Mcal Gross energy intake level the losses in feces, urine, methane and heat production was 45.82%, 5.40%, 4.31% and 33.01%, respectively, and net energy retention for milk production was 11.43%. The gross efficiency of conversion of metabolic energy ME for milk production was 35.69% and the net efficiency of conversion of ME for milk production was 39.56%.

## Introduction

Efficiency of utilization of energy for milk production is governed by a variety of factors; specifically ration composition, environmental temperature, and stage of lactation.^1^ No studies have been reported to determine the efficiency of energy utilization in lactating crossbred cattle regarding the system of husbandry in India. Hence, the present study was aimed to determine the efficiency of energy utilization for milk production in lactating crossbred cattle when they were fed with concentrate and mixed roughage (berseem + wheat straw) along with the replacement of dicalcium phosphate with diammonium phosphate in the mineral mixture.

## Materials and Methods

The present experiment was conducted on eighteen lactating crossbred cattle of approximate body weight (375.39 ± 23.43 kg), milk yield, parity and stage of lactation which divided into three groups of six animals each. In the experimental groups, the di-calcium phosphate (DCP) in the mineral mixture (T_1_ as control) was replaced with 50% DAP (T_2_) and 100% DAP (T_3_) (Table1). The required amount of urea was incorporated in the mineral mixture (T_1_ and T_2_) to keep the rations isonitrogenous. Different amounts of limestone were added to all the diets to maintain the identical calcium content. The animals were fed a calculated quantity of balanced ration to fulfill their nutrient requirements according to Indian Council of Agricultural Research (ICAR) standards.^2^

**Table 1:** Ingredient composition of mineral mixtures.

Ingredients	DCP (100%)	DCP (50%) + DAP (50%)	DAP (50%)
DCP	31.34	15.67	-
DAP	-	15.67	31.34
LSP	21.18	33.15	45.12
Common Salt	21.66	21.66	21.66
TM*	1.87	1.87	1.87
Urea	14.26	7.13	-
Filler	9.67	4.84	-
Ca %	15.34	15.34	15.34
P %	6.58	6.42	6.26

* Trace mineral contained cobalt chloride 40g, copper sulfate 240 g, ferrous sulfate 780 g, manganese sulfate 780 g, sodium selenite 8 g and potassium iodide 24 g.

Clean drinking water was offered to the animals twice daily. The ration scheduled was adjusted weekly on the basis of the milk production of the crossbred cattle. All the animals were offered weighed amounts of mixed roughage (berseem + wheat straw). Concentrate allowance was offered in two portions, one in the morning milking (3:00 AM) and the other in the afternoon milking (3:30 PM). The concentrate mixture consisted of 40 parts crushed maize, 22 parts wheat bran, 35.5 parts mustard cake, 2 parts mineral mixtures and 0.5 part common salt. Milk records were kept for individual cows throughout the experimental period.

Animals were fed experimental rations for 120 days inclusive of seven days metabolic trial, which was conducted at the end of the trial period. Feces and urine were quantitatively collected and were preserved for further analysis. Aliquots of milk were taken during morning and afternoon for each animal. Feces, urine, feeds, residues and milk were analyzed for proximate constituents according to A.O.A.C.^3^ The fat content of milk was determined in Soxhlet apparatus.^4^ The data obtained during experiment were analyzed by using randomized block design method as described previously.^5^

GE of a feed was calculated from its chemical composition as per the formula suggested by Ewan:^6^

*GE (kcal kg* ^-1^ *) = 4143 + (56Χ% EE) + (15Χ% CP) - (44Χ% ash)*

Digestible energy (DE) was calculated from the Total digestible nutrients (TDN) value obtained (1g TDN= 4.4 kcal DE). Urine (10%) and methane (8%) losses were calculated from DE.

The gross efficiency of milk production was calculated presuming 1kg 4% Fat Corrected Milk (FCM) contained 750 Kcal and 1 kg TDN contained 3600 kcal ME.^7^ The gross efficiency of ME of milk production was calculated as follows:

Grossefficiency of milkproduction=750 FCM (kg)3600TDN1(kg)×100

The net efficiency of milk production was calculated by subtracting TDN or ME utilized for the maintenance from total energy intake. 

## Results

The chemical composition of the experimental diets (concentrate mixtures) and mixed roughage offered to the experimental animals are presented in Table 2. Due to replacement of DCP at 50% in T_2_ and 100% in T_3_ diet, the chemical composition in respect of CP, EE, CF, ash, Ca and P content did not vary as compared to the control (T_1_). 

The digestibility coefficient of various organic nutrients is shown in Table 3. There was no significant difference in the digestibility of DM, CP, EE and NFE of experimental diets.

Intake of all the nutrients (Table 4) was similar in the control (T_1_) and the experimental groups (T_2_ and T_3_). During the experimental period the animals showed very little change in body weight. 

**Table 2:** Chemical composition of concentrate mixtures and mixed roughage on DM basis (%).

**Particular**	**Concentrate mixtures**			**Mixed roughage**
	DCP (100%)	DCP (50%) + DAP (50%)	DAP (50%)	
DM	92.96	92.48	93.01	39.86
CP	19.96	19.88	20.15	06.24
EE	4.39	4.48	4.74	02.23
CF	6.21	6.76	6.54	30.29
Ash	11.08	11.57	11.76	09.76
NFE	58.36	57.31	56.81	51.48
Ca	1.09	0.91	1.02	0.34
P	0.82	0.77	0.86	0.22

All the three diets (T1, T2 and T3) were comparable in dry matter intake and digestibility of organic nutrient, hence the data were pooled for eighteen lactating crossbred cattle and the distribution of GE and the efficiency of utilization of energy was calculated by a factorial method.

Percentage distribution of gross energy in feeds, feces, urine, methane, milk and heat production and tissue deposition is presented in Table 5. The energy of heat production and tissue deposition was calculated as gross energy consumed, which was not excreted in feces, urine, methane or milk.

**Table 3:** Digestibility coefficient of organic nutrients

**Organic Nutrients**	DCP (100%)	DCP (50%) + DAP(50%)	DAP (50%)
DM	58.57 ± 1.70	60.46 ± 2.90	61.51 ± 2.00
CP	60.50 ± 1.85	61.63 ± 3.67	63.19 ± 2.06
EE	51.85 ± 2.57	54.64 ± 3.55	54.20 ± 2.12
CF	74.22 ± 1.19	76.12 ± 1.25	77.89 ± 1.15
NFE	73.12 ± 1.00	73.84 ± 1.90	74.66 ± 1.28

**Table 4:** Daily nutrient intake, live weight changes and milk production in lactating crossbred cattle.

**Particulars**	**DCP (100%)**	**DCP (50%) + DAP (50%)**	**DAP (50%)**	**Average**
DMI (g kg^-1^ W^0.75^)	130.94 ± 7.55	129.90 ± 7.64	126.91 ± 4.84	117.78
DCP (g)	623.71 ± 26.92	617.51 ± 34.96	625.28 ± 29.05	622.16
TDN (kg)	6.05 ± 0.26	5.53 ± 0.30	5.43 ± 0.20	5.67
ME (Mcal)	21.07 ± 1.49	20.01 ± 1.40	20.66 ± 1.38	20.58
Gain/loss(g d^-1^)	78.44 ± 22.53	41.76 ± 34.51	25.99 ± 18.00	48.73
4% FCM production (kg d^-1^)	9.85 ± 0.19	9.55 ± 0.13	9.77 ± 0.16	8.58

**Table 5:** Distribution of gross energy

**Energy**	**GE (Mcal)**	**FE (Mcal)**	**DE (Mcal)**	**UE (Mcal)**	**Methane (Mcal)**	**Milk (Mcal)**	**HP & TD (Mcal)**	**NE**_Milk _ **(Mcal)**
Distribution	46.07	21.11	24.96	2.49	1.99	20.48	15.21	5.27
% of GE	100	45.82	54.17	5.40	4.31	44.45	33.01	11.43

HP & TD = Heat production and tissue deposition.

## Discussion

The gross efficiency of ME for milk production was 35.69%, which is within the range of 19.10 to 38.60% for various types of roughages.^8^ Efficiency of milk production increases from 18.54 to 20.11% with the complete feed as compared with conventional type of feeding system.^9^

The net efficiency of ME for milk production was 39.56%, which is similar to that (37.57%) during mid-lactation.^10^

In the present study out of 46.07 Mcal GE intake level, the losses in feces, urine, methane and heat production was 45.82%, 5.40%, 4.31% and 33.01%, respectively, leaving behind a net energy retention for milk production as 11.43%. Various losses at the same GE intake level (53.1Mcal) as 37.00% in feces, 2.30% in urine, 5.50% in methane, 14.80% in milk production and 40.40% in heat production and tissue deposition in cross bred cows.^8^ The lower fecal loss in the present study may be due to higher DM digestibility. The higher heat production in our study may be due to higher roughage to concentrate ratio (82:18) compared to 50:50 ratio.^8^ Reportedly, higher (52.24%) during early and lower (29.50%) in late stage of lactation. The efficiency of utilization of energy for milk production is governed by a variety of factors such as ration composition, environmental temperature and stage of lactation.^1^

High environmental temperature caused a significant decrease in efficiency of energy utilization for milk production.^11^ Similarly, low efficiency of energy utilization for milk production in our experiment was due to the high environmental temperature (average 38.31˚C) in June during the metabolic trial period.

It was concluded that out of 46.07 Mcal GE intake, the losses in feces, urine, methane and heat production in addition with tissue deposition were 45.82%, 5.40%, 4.31% and 33.01%, respectively, and the net energy retention for milk production was 11.43%. 
